# The Factors and Relationships Influencing Forest Hiking Exercise Characteristics after COVID-19 Occurrence: At Seoul Metropolitan Area and in Hikers’ 20s and 30s

**DOI:** 10.3390/ijerph192416403

**Published:** 2022-12-07

**Authors:** Bobae Lee, Poungsik Yeon, Seoncheol Park

**Affiliations:** 1 Department of Information Statistics, Chungbuk National University, Cheongju 28644, Republic of Korea; 2 Department of Forestry, Chungbuk National University, Cheongju 28644, Republic of Korea

**Keywords:** COVID-19, urban mountains, exercise recorded data, hiking exercise characteristics, Millennials and Generation Z, linear mixed model

## Abstract

The growing interest and usage of green space during the pandemic relates to why greenery increases enjoyment of daily life and alleviates troubles arising from infectious periods. However, it is necessary to understand what reactions to the degree of COVID-19’s spread were expressed in hiking exercise characteristics (hiking frequency, actual movement distance, average speed, total exercise time). To understand the progress of forest usage behavior during the pandemic, we analyzed factors influencing hiking exercise characteristics and relationships between those factors and hiking exercise characteristics. Hiking record data were obtained (2019–2020) from the workout app “Tranggle” pertaining to hikers in their 20s and 30s who visited the 13 mountains in the Seoul Metropolitan Area most frequently. The differences in hiking exercise characteristics (2019 data subtracted from 2020) were linked with factor data that could be related to them, including the degree of COVID-19’s spread ($R_{t}$). To explore relationships between hiking exercise characteristics and factors, we developed four models with a linear mixed model. We found that $R_{t}$, week, weekdays/weekends, and ${\mathrm{PM}}_{10}$ contributed to explaining the hiking exercise characteristics’ differences. As a result of this analysis, the degree of COVID-19’s spread ($R_{t}$) affected hiking frequency; the week affected hiking frequency and actual movement distance; weekdays or weekends affected hiking frequency, actual movement distance, average speed, and total exercise time; and ${\mathrm{PM}}_{10}$ affected hiking frequency. These findings indicate that hiking was an alternative way for those looking for a new strategy to replace lost opportunities for physical activity. Therefore, we conclude that it is necessary to induce the usage of green space so that many people can take advantage of the functions and benefits of greenery, which stood out during the pandemic era.

## 1. Introduction

After 2020, when COVID-19 caused a global pandemic, the measures to prevent the spread of COVID-19 have basically changed the way people participate in personal and public activities [1]. Because of the pandemic, unneeded trips and international tourism diminished rapidly and domestic needs grew [2], centered on green spaces in areas such as suburbs of cities and rural regions of the country [3,4]. Likewise, Korea also increased people’s attention and usage of small-scale outdoor activities in natural spaces that could comply with quarantine policies outside of a hermetic, dense, closed environment. Moreover, the mention of natural green space online rose compared with before COVID-19 [5,6,7,8].

Surpassing the Alpha, Beta, Delta, Omicron variants, etc., the significant variance that seemed to have high pathogenicity in the early pandemic now results in reduced symptoms and lowered mortality, and many countries such as the UK, the USA, France, Germany, Denmark, etc., have relaxed their quarantine systems [9]. However, Anthony Fauci and the WHO noted that the present state of the pandemic does not mean COVID-19 has been stamped out [10,11]. In addition, sustaining change in climate and land usage stimulates zoonosis because it generates the chance for disease to spread in the shifting habitats of wild animals [12]. Moreover, it is seen that infectious diseases seem to be transmitted easily without difficulty because we have become more highly connected owing to several factors, such as transportation technology, globalization, etc. Infectious diseases can be expected to disseminate across a vast area, and the occurrence cycle is short. Therefore, it is necessary to think back on the effect of COVID-19 on during the turnover the pandemic period to an endemic one. After COVID-19, what change was aroused in the usage of forests? We need to identify what factors affect hiking exercise characteristics and what the relationships are between these factors and hiking exercise characteristics. Through this processing, we could comprehend the social change phenomena due to infectious diseases and realize causal relationships. It is helpful because it properly informs what aspects of the forest usage should be considered to quickly decrease health problems that could occur in a crisis from infectious diseases.

During the pandemic, people started paying attention to the benefits of forests on health and welfare. As a result of measures such as social distancing, working from home, and social distancing education (e.g., online classes, remote video classes), on the whole, physical activity decreased [13,14], and because of this, body and mind problems began to occur in a narrow sense and threats to health welfare occurred in a broader sense. Physical activity in green spaces or living nearby greenery improves mental health, such as easing depression, reducing stress, and mitigating mental health burdens [15,16,17,18,19] because of the close link between physical and health [19]. Visiting green spaces such as parks, urban forests, and national parks supports well-being [20], cushions the decrease in leisure-time physical activity [21], and also encourages the social cohesion of residents in high-density urban neighborhoods [22]. In the studies mentioned above, the interest and usage of green space that can comply with quarantine rules and be less restricted in activity explains that greenery could be utilized as a way to enjoy daily life and as a way to solve problems arising from infectious circumstances. Therefore, understanding the usage of green spaces and physical activity changes empirically prepares us for the subsequent waves of COVID-19 and presents strategies to cope with new contagious diseases. This understanding is also crucial because it could present guidelines based on evidence for public policy and individual selection.

This study demonstratively analyzes the usage behavior of forests according to the degree of COVID-19’s spread since the beginning of the COVID-19 pandemic. We are using recorded data on the change in physical activity characteristics (hiking frequency, actual movement distance, average speed, total exercise time) due to the effects of social circumstances. We obtained data from one year before to one year after COVID-19’s occurrence to examine changes in long-term physical activity characteristics. The exercise dataset, which is recorded of one’s own accord without artificial intervention, can be seen to be the result of natural experiments and provides a chance for causal inference [23]. In addition, analyzing the mobility of people is an excellent strategy to progress the speed and accuracy of fundamental data offered for social circumstances [24]. With this background, observing hiking activity and finding factors influencing it and the relationships between those factors, including the degree of COVID-19’s spread and the hiking characteristics, can help decision making for outdoor activities in the infectious era. These results provide the primary data for better public policy making in similar crises.

This study aims to find factors and the relationship influencing hiking exercise characteristics (hiking frequency, actual movement distance, average speed, total exercise time) concerning the degree of COVID-19 spread and other related factors (e.g., week, weekdays or weekends, ${\mathrm{PM}}_{10}$, average daily temperature, etc.). This paper therefore addresses the following research questions:

1. What factors have affected the change in hiking exercise characteristics?
2. What kind of relationship exists between these factors and hiking exercise characteristics?

## 2. Materials and Methods

### 2.1. Study Design and Study Sites

This study’s procedure is as follows: First, the study period and sites were restricted because of the volume, velocity, and variety of the accumulated digital data. The period, age, and location range were selected based on prior studies and forest use statistics. Millennials and Generation Z (anyone born between 1984 and 1996 is considered a Millennial, and anyone born between 1997 and 2010 is considered a Generation Z [25]) tend to feel a sense of accomplishment through exercise in COVID-19 circumstances [26]. Hiking is the way to re-building the crumbling daily life in Millennials and Generation Z [25]. The young generation who did not hike a lot in the past [27] has noticeable hiking usage differences before and after the pandemic [28,29,30]. In addition, the visit ratio was high in urban forests [31,32,33] and comprised 50.4 percent of the population living in the Seoul Metropolitan Area [34]. Lastly, we selected data from the beginning of 2019 to the end of 2020 to compare the exercise characteristics before the COVID-19 outbreak with those after that. Second, we consulted with a company that operates a workout app (Tranggle); after that, we cut down the study range to the top 13 mountains in terms of the frequency of visits and were provided with data on users in their 20s and 30s through approval procedures for data use. Third, data preprocessing was performed to appropriately process data, such as extracting parts of the data, dividing them by type, or combining multiple points of data [35]. Fourth, before the data were compared to the probabilistic model, flexible research was conducted through exploratory data analysis [36] (this is the follow-up study that Lee and Yeon conducted; a detailed description of the exploratory data analysis results may be found in [37]). Fifth, we want to know the general hiking exercise characteristics, not specific users within the study’s age and site ranges. Thus, we implemented a linear mixed model. This model can identify, separately and simultaneously, fixed (model average trends, such as $R_{t}$, week, weekdays or weekends, ${\mathrm{PM}}_{10}$, temperature in this case) and random model effects (model the extent to which these trends vary across levels of some grouping factor, in this case, mountains) [38].

### 2.2. Study Site

**Figure 1:**
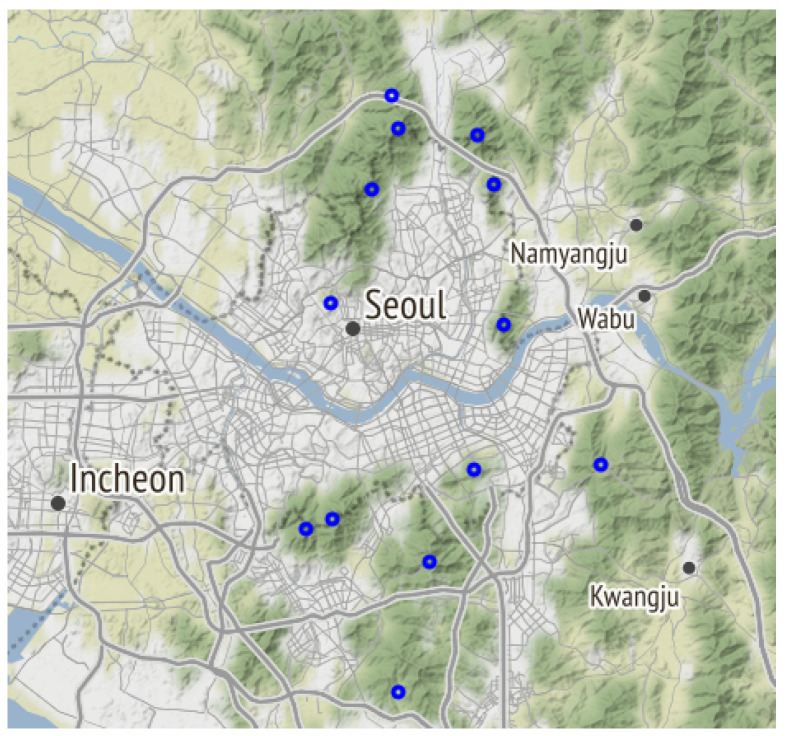
The cities in Metropolitan Area (black circles) and the study sites (blue circles).

The most popular 13 mountains in the Seoul metropolitan area for hikers in their 20s and 30s in 2019 and 2020, mostly located in the outskirts of Seoul, were peri-urban forests that form the framework of the city. The study sites were: Acha Mountain, Gwanak Mountain, Gwanggyo Mountain, Inwang Mountain, Bulam Mountain, Suraksan Mountain, Dobong Mountain, Sapae Mountain, Daemo Mountain, Cheonggye Mountain, Bukhan Mountain, and Namhan Mountain. All or parts of 9 of these mountains are within Seoul or forest areas in affluent suburban areas outside of Seoul (Figure 1). In addition, eight of these mountains are interconnected by the Seoul Dulle-Gil that circles the city of Seoul, connecting suburbs and the center with prominent mountains. The heights of the 13 mountains were 287–836 m, with a mean elevation of 538 m.

### 2.3. Data Collection and Variable Selection

#### 2.3.1. The User Recording Data

Beagle, the operator of the exercise app “Tranggle” collected big data specialized for outdoor exercise and activities and provides various services and information for the user, for example, a Customized Hiking and Trekking Information Guide [39]. The data used in this study were provided through approval procedures after consulting with the relevant company. The period of the data is from January 2019 to early December 2020. The number of data sets is 142,806 for 2019, 194,698 for 2020, and the total number of data sets is 337,504.

#### 2.3.2. $R_{t}$ (Time-Varying Reproduction Number)

**Figure 2:**
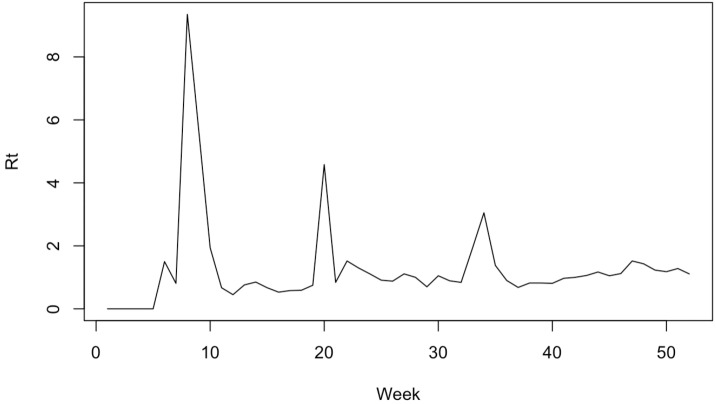
The Korea $R_{t}$ in 2020 by week.

Reproduction number (R) is the average number of people a patient infects during a contagious period. If R > 1, the epidemic continues; if R < 1, the incidence decreases; if R = 1, it means the disease is endemic [40]. $R_{t}$ (time-varying reproduction number) is the average infectivity of the population at a specific point in time and is useful for estimating the number of new infections on a specific day, retrospectively or in real-time [41]. $R_{t}$ is a valuable indicator of whether the disease is spreading or not, and it is used as judgment data for setting the direction of quarantine measures [42]. So, to measure the degree of COVID-19’s spread and compare it with the following infectious circumstance and other national circumstances, we used $R_{t}$ data. These data were obtained from Yoo et al. [41] at the Korea Centers for Disease Control and Prevention. (Figure 2).

#### 2.3.3. The Others

Weather factors affect hiking related to outdoor activity characteristics. To separate before and after hiking exercise differences from COVID-19 effects, it is necessary to compare seasonal atmospheric factors with the degree of COVID-19 spread ($R_{t}$). The Korea Meteorological Agency provided weather observation data such as average temperature, daily precipitation, average wind speed, etc. When implementing the preliminary examination to find appropriate values, the average wind speed was not statistically significant; daily precipitation data had many zero values, so we thought it was not robust to outliers. After discussion, it was decided to remove average wind speed and daily precipitation variables according to the principle of parsimony. Average daily temperature and ${\mathrm{PM}}_{10}$ were included as explanatory variables. Particulate matter (PM) is made up of particles (tiny pieces) of solids or liquids that are in the air [43]. PM impacts human health and the climate. The most significant PM which relates to health are particulates of diameter less than or equal to 10 nanometers, usually designated as ${\mathrm{PM}}_{10}$ [44].

### 2.4. Analysis

#### 2.4.1. Data Preprocessing

The data provided have 22 variables, including classification labels, actual movement distance, average speed, maximum speed, the length of time spent hiking, track name, and acquisition date. We used these variables to create derived variables. Hiking was extracted from classification labels. As acquisition dates were divided into the year and day of the week, “Year”, “week”, and “weekdays/weekends” categories were created. In addition, a “Mountain name” variable was created according to each file name provided. Afterward, final data were processed by checking and removing missing values, outliers, and weeks in which data for all 13 mountains were not included. Summary statistics of final data are displayed in Table 1. To analyze the data, we computed the weekly temperature average, ${\mathrm{PM}}_{10}$, exercise data, $R_{t}$, etc. Moreover, to observe hiking characteristics pattern changes before and after the COVID-19 outbreak, hiking groups in the combined data were rearranged by mountain name, year, weeks, and weekdays or weekends. Weeks without data (weeks 49–52) were excluded.

#### 2.4.2. Modeling

This study was fit to a model using a linear mixed model to find the changing relationship between the exercise characteristics (hiking frequency (HF), actual movement distance (AMD), total exercise time (TET), and average speed (AS)) and the degree of COVID-19 spread. The square root transformation is used for hiking frequency because heteroscedasticity was suspected when analyzing the residuals. The best model is selected based on the Akaike information criterion (AIC), the most popular model selection method. The linear mixed model was analyzed with the gam function of R’s mgcv library [45]. The linear mixed model can be described as

(1) $$Y_{ij}\;=\;\beta_{0}+\beta_{1}R_{ij}+\beta_{2}W_{ij}+\beta_{3}D_{ij}+\beta_{4}P_{ij}+\gamma_{1j}+\epsilon_{ij}$$

where $Y_{ij}$ = hiking exercise characteristics (number of hiking exercise characteristics, actual movement distance, average speed, total exercise time); $\beta_{0}$ = each hiking exercise characteristic’s expected mean; $R_{ij}\;=\;R_{t}$ values; $\beta_{1}$ = the fixed $R_{t}$ values effect; $W_{ij}$ = weeks; $\beta_{2}$ = the fixed effect of weeks; $D_{ij}$ = whether the acquisition date is weekends ($D_{ij}$ = 1)/ weekdays ($D_{ij}$ = 2); $\beta_{3}$ = the fixed effect of whether the acquisition date is weekdays/weekends; $P_{ij}\;=\;PM_{10}$; $\beta_{4}$ = the fixed ${\mathrm{PM}}_{10}$ effect; $\gamma_{1j}$ = the random site (mountain) effect; $\gamma_{1j}∼N\left(0,\sigma_{\gamma }^{2}\right),N\left(0,\sigma_{\gamma }^{2}\right)$ means a normal distribution with mean zero and variance $\sigma_{\gamma }^{2}$; $\epsilon_{ij}$ = independent random error variables, where $\frac{\epsilon }{\sigma }$ follows the t-distribution with degree of freedom ν i = 1, …, and Hiking frequency, j = 1, …(number of observations of each mountain). In this model, $\beta_{0},\beta_{1},\beta_{2},\beta_{3},\beta_{4},\sigma_{\gamma }^{2}$, and μ are parameters to be estimated. After fitting a model for each hike exercise characteristic, the selected parameters could best explain useful variables in the model using AIC.

## 3. Results

### 3.1. Hiking Frequency (Figure 3)

**Figure 3:**
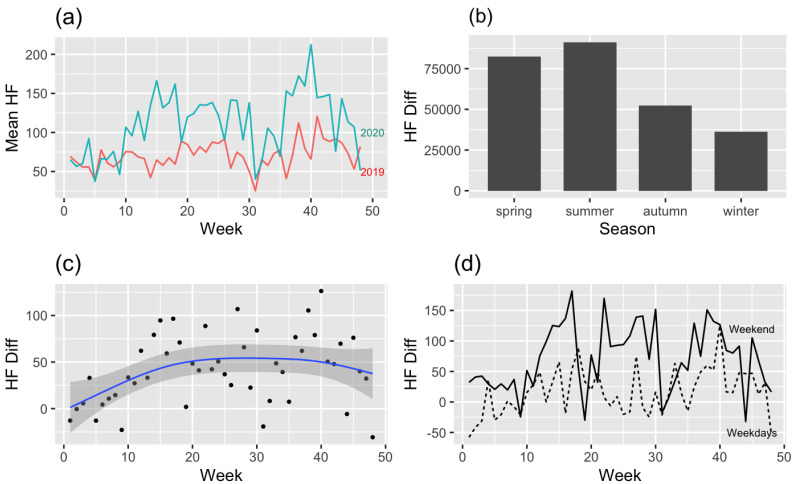
(**a**) Mean hiking frequency (2019–2020) by week, (**b**) Hiking difference count by season, (**c**) regression model (linear mixed model) graph of hiking frequency difference (2019–2020), and (**d**) difference of hiking frequency between weekdays and weekends (2019–2020).

Hiking frequency in 2020 compared to 2019 before the COVID-19 outbreak was high in the order of Summer, Spring, Autumn, and Winter (Figure 3a,b). The season criterion refers to [46]. The trend line (Figure 3c) more clearly shows evidence of the above argument. The difference between 2019 and 2020 steadily advanced at 20 weeks and was maintained for 20–40 weeks, and the difference became less pronounced after 40 weeks. Note the difference between weekdays and weekends (Figure 3d), with more hiking on weekends than on weekdays for the extended period. In the modeling of hiking frequency differences within two years, the difference is affected by $R_{t}$ (Parametric coefficients: −0.20, *p* < 0.001), weekdays or weekends (Parametric coefficients: −2.00, *p* < 0.001), and ${\mathrm{PM}}_{10}$ (Parametric coefficients: −0.02, *p* < 0.001) (Table 2). For a better understanding, the most common situations can be substituted for the derived model of hiking frequency as follows: $\sqrt{Y_{ij}}\;=\;\beta_{0}+\beta_{1}R_{ij}+\beta_{3}D_{ij}+\beta_{4}P_{ij}+\gamma_{1j}+\epsilon_{ij}$, where the mean $R_{t}$ is 1.079 and the mean ${\mathrm{PM}}_{10}$ is 77.99 from these data. It can be interpreted that the hiking frequency of hikers in their 20s and 30s who climb mountains in the metropolitan area has increased 106.71 times on average since the outbreak of COVID-19, with an $R_{t}$ of 1.079 and a ${\mathrm{PM}}_{10}$ of 77.99. Under the same conditions, the frequency was improved by 172.66 times on weekends. The week did not statistically significantly affect the hiking frequency, so it was excluded from this model.

### 3.2. Actual Movement Distance (Figure 4)

**Figure 4:**
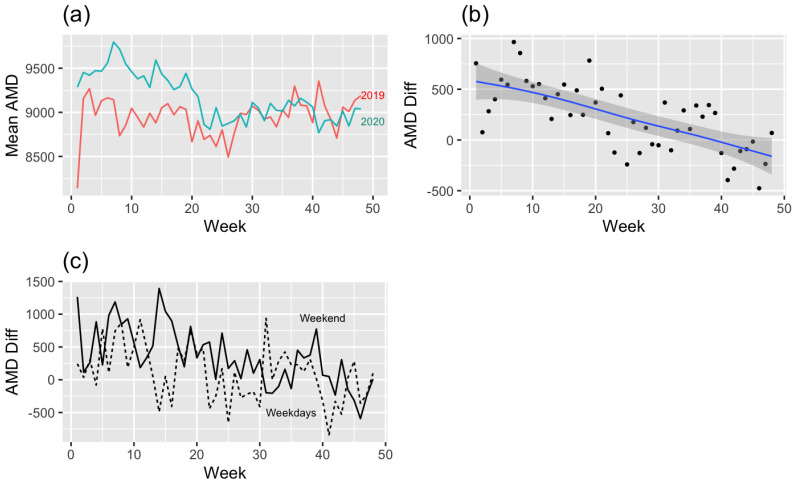
(**a**) Mean actual movement distance (2019–2020) by week, (**b**) regression model (linear mixed model) graph of actual movement distance difference (2019–2020), and (**c**) difference of actual movement distance of weekdays or weekends.

Figure 4a shows actual movement distance differences (2019–2020). It shows an apparent difference between weeks 0 and 30 compared with 2019, before the outbreak of COVID-19. The trend line (Figure 4b) more clearly shows evidence of the above argument. The difference between 2019 and 2020 was prominent in the early days of COVID-19 and, after that, the difference steadily dwindled. Looking at the difference between weekdays and weekends (Figure 4c), the actual movement distance on weekends is longer than on weekdays throughout most periods, except for specific weeks. In the modeling of actual movement distance difference within two years, the difference is affected by week (Parametric coefficients: −17.00, *p* < 0.001) and weekdays or weekends (Parametric coefficients: −235.00, *p* < 0.01) (Table 2). For a better understanding, the most common situations can be substituted for the derived model of actual movement distance as follows: $Y_{ij}\;=\;\beta_{0}+\beta_{2}W_{ij}+\beta_{3}D_{ij}+\gamma_{1j}+\epsilon_{ij}$. It can be interpreted that the actual movement distance of hikers in their 20s and 30s who climb mountains in the metropolitan area has increased 126.99 m on average since the outbreak of COVID-19, with an $R_{t}$ of 1.079, a ${\mathrm{PM}}_{10}$ of 77.99, and a week mean of 24.53 from these data. Under the same conditions, it increased to 361.99 m on the weekend.

### 3.3. Average Speed (Figure 5)

**Figure 5:**
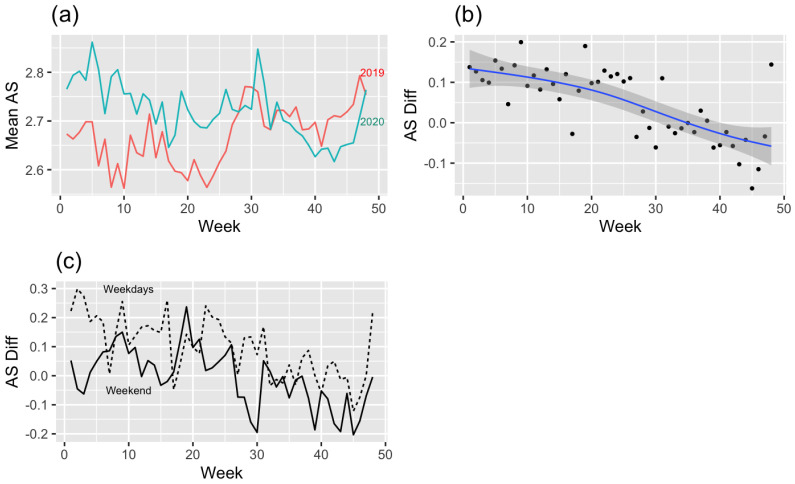
(**a**) Mean average speed (2019–2020) by week, (**b**) regression model (linear mixed model) graph of average speed difference (2019–2020), and (**c**) difference in the average speed of weekdays or weekends (2019–2020).

Figure 5a shows the average speed difference (2019–2020). The difference has positive values at weeks 0–30, then it is changed to negative values. The trend line (Figure 5b) more clearly shows evidence of the above argument. The difference between 2019 and 2020 was prominent in the early COVID-19 period; after that, the difference steadily reduced and became slower compared to 2019 after 35 weeks. Looking at the difference between weekdays and weekends (Figure 5c), the average speed on weekdays is faster than on weekends throughout most of the period, except for specific weeks. In the modeling of the difference in average speed within two years, the difference is affected by weekdays or weekends (Parametric coefficients: 0.03, *p* < 0.001) (Table 2). For a better understanding, the most common situations can be substituted for the derived model of average speed as follows: $Y_{ij}\;=\;\beta_{0}+\beta_{3}D_{ij}+\gamma_{1j}+\epsilon_{ij}$. It can be interpreted that hikers in their 20s and 30s who climb mountains in the metropolitan area have increased their hikes by 126.99 m on average since the outbreak of COVID-19, with a weekly mean of 24.53. It can be interpreted that after the outbreak of COVID-19, the average speed in the 13 mountains in the Seoul Metropolitan Area which were visited by most people in their 20s and 30s, increased by 0.06 m/s on average. Under the same conditions, the speed increased by 0.03 m/s on the weekend. The week also statistically significantly affected the average speed, but the coefficient estimate was zero, so it was excluded from this model.

### 3.4. Total Exercise Time (Figure 6)

**Figure 6:**
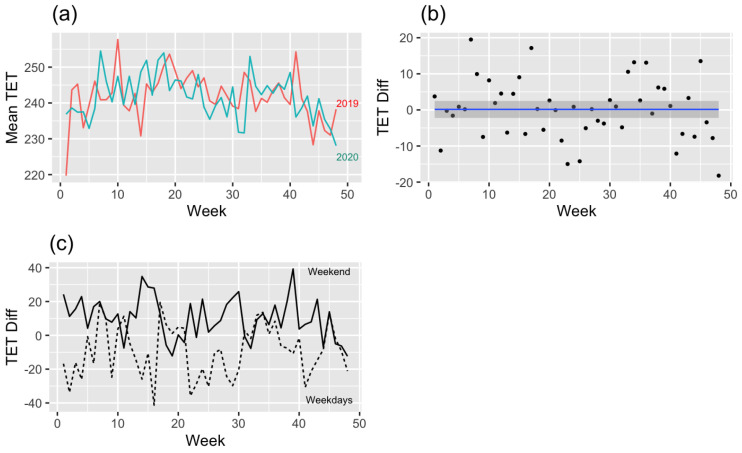
(**a**) Mean total exercise time (2019–2020) by week, (**b**) regression model (linear mixed model) graph of the total exercise time difference (2019–2020), and (**c**) difference in total exercise time of weekdays or weekends (2019–2020).

Figure 6a shows the total exercise time difference (2019–2020). The trend line (Figure 6b) more clearly shows evidence of the above argument. It is observed that there is no difference between 2019 and 2020 in total exercise time. Looking at the difference between weekdays and weekends (Figure 6c), the total exercise time on weekends was high compared to 2019 for the longest period. In the modeling of the total exercise time differences within two years, the difference is affected by weekdays or weekends (Parametric coefficients: −18.59, *p* < 0.001) (Table 2). For a better understanding, the most common situations can be substituted for the derived model of total exercise time as follows: $Y_{ij}\;=\;\beta_{0}+\beta_{3}D_{ij}+\gamma_{1j}+\epsilon_{ij}$. It can be interpreted that hikers in their 20s and 30s who climb mountains in the metropolitan area have increased their exercise time by 7.33 min on average since the outbreak of COVID-19 on weekdays. Under the same conditions, it grew by 25.92 min on the weekend.

## 4. Discussion

This study aimed to investigate the factors and relationships influencing the hiking exercise characteristics after the COVID-19 outbreak, focusing on people in their 20s and 30s who hiked the mountains in the urban and peri-urban Seoul Metropolitan Area. These results indicate that the degree of COVID-19’s spread ($R_{t}$) affected hiking frequency. It was also found that various factors differently influence the hiking exercise characteristics and the relationships between related factors and hiking exercise characteristics.

Hiking frequency increased until week 40; however, total exercise time has stayed the same since the first year of the pandemic. Actual movement distance and average speed were prominently high in the early pandemic, but a reduction was observed after that. The following were observed: Since COVID-19 occurence, hiking frequency has increased and hiking participation was different depending on the degree of COVID-19’s spread, but time spent hiking remained consistent regardless of spread. Actual movement distance and average speed seemed to be associated with social circumstance; the difference in actual movement distance from the previous year’s was high until about week 20 (Figure 4a). In the case of the increase in average speed, it became more rapid after week 20 (Figure 5b). The high-strength social distancing, such as refraining from going out, was implemented during this period. In addition, the average speed in 2020 soared, overlapping with the time of the curve that confirmed cases increased sharply. Comprehensively, it could be interpreted that the MZ generation was looking for an alternative to lost opportunities for incidental or non-incidental physical activity as anxiety was high as a result of having no information about a new virus. They were flexible, coped with social circumstances, and kept hiking. Hiking was one of the alternative modes of exercise they selected. These changes, such as the kind of exercise and patterns arising from COVID-19, were also seen in the age groups [47,48] and with time, showing that individuals learn to adapt to situations in healthy and positive ways [48].

Di Sebastiano et al. [49] looked at ten weeks before and after COVID-19’s occurrence through the data of the national physical activity tracking app (PAC app) that recorded physical activity strength by heart rate and number of steps walked. They stated that moderate-to-vigorous physical activity, light physical activity, and total steps significantly decreased immediately following the declaration of the pandemic. However, six weeks later, moderate-to-vigorous physical activity had returned to prepandemic levels. A Community Health Survey in Korea [13] reported that low-strength exercise (walking) and moderate physical activity declined after the pandemic outbreak. The results of the Community Health Survey in Daegu (Korea) [50] showed that the people who do not practice moderate to vigorous physical activity, residents in urban areas, those with high health anxiety from COVID-19 infection, and those with perceived depression and obesity had decreased physical activity compared to before the COVID-19 pandemic. In those 20–40 years old, the level of physical activity also relatively significantly decreased; they said it is because they are the generation who engaged in active physical activity prepandemic. Wilke et al. [51] performed a survey in 14 countries and reported that compared to the prerestrictions period, overall self-reported PA declined by 41% (vigorous physical activity) and 42.2% (vigorous physical activity). Reductions were higher for working people, younger and older people, and previously more active people but were similar between men and women. In this context, Jin and Kim [50], Hargreaves et al. [52], and Tornaghi et al. [53] said that intervention in the activity status of individuals prelockdown needs to be taken into account.

Given that COVID-19 presented vulnerabilities in the structure of society [54,55], counteracting COVID-19 was, in addition to managing the virus, moreover about understanding the crucial social-economic crisis arising from infectious diseases and counteracting them [56]. Physical activity has decreased due to restrictions established after the pandemic’s occurrence. The timeline was different in each country, but interest in and usage of green spaces grew in this era evenly. During the pandemic, greater use forestry space, such as urban green space and green infrastructure, could be considered an active, reasonable detour strategy. It shows the possibility of utilization and the importance of forestry space in an epidemic of infectious diseases. Based on our results, policy guidelines for o mitigating physical activity loss to preserve health are the following: First, people are flexibly coping with the degree of COVID-19’s spread. However, providing programs such as self-guided therapeutic programs and app content when the spread of COVID-19 is high is needed to encourage continuous participation. Second, once they start hiking, people take a certain amount of time for exercise regardless of the degree of COVID-19’s spread, so it is necessary to guide participants through the connecting forestry space with other resources in a community to increase ease of access to hiking exercise.

Prior studies of the usage behavior of forests have been surveyed, either focused on parts of regions or peak seasons [27,31,32,33]. However, surveys lacked information such as date from a broad area, time series data on usage behavior, and specific hiking characteristics [57]. We used self-recorded data collected from the exercise app “Tranggle”. We could therefore look at a wider region and over a longer term. However, we still have limitations in the sense that we only looked at two generations and at one metropolitan area due to the policies of the company with regard to sharing research data. It is necessary to follow-up with studies concerning other ages, gender differences, and specific vulnerable groups that showed decreased physical activity and interest.

These results could be provided to individuals and public institutions for better selection of physical and psychological aspects under social-distancing circumstances.

## 5. Conclusions

We investigated factors and relationships influencing hiking exercise characteristics after the pandemic outbreak. We found that the degree of COVID-19’s spread was affected by certain characteristics and what the relationships were between influencing factors and hiking exercise characteristics. Hiking was one of the alternatives to losing opportunities for physical activity. Moreover, the people who use “Tranggle” in their 20s and 30s actively participated in hiking to cope with the spread of COVID-19. It is necessary to provide the various usage program to prepare for the periods the degree of COVID-19 spread is high for continuous participation. Further, to induce hiking exercise, increase ease of access through various ways such as relating the resources.

## Figures and Tables

**Table 1:** Comparison of representation values for each characteristic.

	Year	Mean	Std	25%	50%	75%	Max
The number of hikers	2019	69	51	32	55	95	394
	2020	108	78	12	25	147	436
Actual movement distance	2019	8978	3911	6436	8526	10,991	32,836
	2020	9156	3893	6606	8672	11,151	32,806
Average speed	2019	3	1	2	3	3	6
	2020	3	1	2	3	3	6
Total exercise time	2019	254	104	177	249	326	793
	2020	251	104	174	245	321	798

**Table 2:** Estimated regression coefficients with their 95% confidence intervals and *p*-values of the linear mixed model.

	Square Root of Hiking Frequency Difference		Actual Movement Distance Difference		Average Speed Difference		Total Exercise Time Difference	
Fixed Effect	Parameter Est (95% CI)	Pr (>|z|)	Parameter Est (95% CI)	Pr (>|z|)	Parameter Est (95% CI)	Pr (>|z|)	Parameter Est (95% CI)	Pr (>|z|)
Intercept	14.09 (13.07, 15.12)	<0.001 ***	779 (−141, 1698)	0.10	0.03 (−0.03, 0.09)	0.32	25.92 (−6.07, 57.90)	0.11
$R_{t}$	−0.20 (−0.29, −0.10)	<0.001 ***	-	-	-	-	-	-
week	0.01 (0.00, 0.03)	0.10	−17 (−23, −10)	<0.001 ***	0	<0.001 ***	−0.25 (−0.51, 0.00)	0.05
weekdays	−2.00 (−2.31, −1.70)	<0.001 ***	−235 (−419, −52)	0.01 *	0.03 (0.02, 0.04)	<0.001 ***	−18.59 (−24.41, −12.78)	<0.001 ***
${\mathrm{PM}}_{10}$	−0.02 (−0.02, −0.01)	<0.001 ***	-	-	-	-	−0.13 (−0.25, −0.01)	0.39
Random effect	95% CI	Std.dev	95% CI	Std.dev	95% CI	Std.dev	95% CI	Std.dev
Name	0.81, 1.9	1.2	1098, 2467	1646	0.068, 0.15	0.1	35, 78	52

*** *p* < 0.001, * *p* < 0.05.

## Data Availability

Not applicable.
